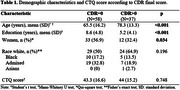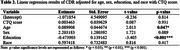# Childhood trauma and cognition status in an elderly sample with a mental disorder

**DOI:** 10.1002/alz70857_107230

**Published:** 2025-12-26

**Authors:** Leonardo Baracat Caria, Salma Rose Imanari Ribeiz, Camila Nascimento Mantelli, Wanessa Santos, Carlos Augusto Pasqualucci, Ricardo Nitrini, Eduardo Ferrioli, Lea T. Grinberg, Renata Elaine Paraizo Leite, Paula Villela Nunes, Claudia Kimie Suemoto

**Affiliations:** ^1^ Faculdade de Medicina de Jundiaí, Jundiaí, Sao Paulo, Brazil; ^2^ Institute of Psychiatry, Faculty of Medicine, University of São Paulo, Sao Paulo, Sao Paulo, Brazil; ^3^ Universidade Federal de São Paulo, Sao Paulo, Sao Paulo, Brazil; ^4^ Department of Pathology, University of São Paulo Medical School, São Paulo, São Paulo, Brazil; ^5^ Department of Neurology, Faculdade de Medicina FMUSP, Universidade de Sao Paulo, Sao Paulo, Sao Paulo, Brazil; ^6^ Division of Geriatrics, Department of Internal Medicine, University of São Paulo Medical School, São Paulo, São Paulo, Brazil; ^7^ Memory and Aging Center, UCSF Weill Institute for Neurosciences, University of California, San Francisco, San Francisco, CA, USA; ^8^ University of British Columbia, Vancouver, BC, Canada

## Abstract

**Background:**

Trauma is the result of distressing events that may affect people in an acute stage or result in chronic consequences. The occurrence of traumatic events throughout life has been considered a risk factor in the development of mental disorders. Although there is evidence that indicates early exposure to trauma may contribute to neurocognitive changes and may contribute to an augmented risk of dementia, there are mixed results. The present study aims to investigate the association between childhood trauma and neurocognitive decline related to dementia.

**Method:**

We included 95 participants from Biobank for Aging Studies of the University of Sao Paulo (BAS‐USP). Subjects were previously diagnosed with a psychiatric disorder using Structured Clinical Interview for DSM‐IV Disorders (SCID) for Axis I, informant part. Childhood trauma and cognitive status were assessed with the Childhood Trauma Questionnaire (CTQ) and the Clinical Dementia Rating (CDR). Demographic and clinical characteristics were compared between individuals with no cognitive decline (CDR = 0) and those with cognitive impairment (CDR > 0) using the Student's t‐test, Mann‐Whitney U test, chi‐square test, or Fisher's exact test, as appropriate. Subsequently, a multivariable linear regression model, adjusted for age, sex, education, and race, was employed to investigate the association of childhood trauma with cognitive status.

**Result:**

Demographics showed an association between CDR scores and the variables age and education, participants in the CDR>0 group were older (*p* <0.001) and had lower education (*p* <0.001) in comparison with the CDR=0 group. Individuals with no cognitive decline (CDR=0) and with cognitive decline (CDR>0) did not differ in CTQ score (*p* = 0.748, table 1). The multivariable linear regression model did not find a statistically significant association between CTQ score and CDR (*p* = 0.931), nor between the other variables (table 2).

**Conclusion:**

Our data does not suggest a relationship between childhood trauma and cognitive decline in a psychiatric sample. Future studies including older participants with and without mental disorders may better elucidate this relationship.